# DNA content of a functioning chicken kinetochore

**DOI:** 10.1007/s10577-014-9410-3

**Published:** 2014-03-15

**Authors:** Susana Abreu Ribeiro, Paola Vagnarelli, William C. Earnshaw

**Affiliations:** 1 Wellcome Trust Centre for Cell Biology, University of Edinburgh, Michael Swann Building, King’s Buildings, Mayfield Road, Edinburgh, EH9 3JR Scotland UK; 2 Department Cellular and Molecular Pharmacology, Howard Hughes Medical Institute, University of California, San Francisco, San Francisco, CA USA; 3 Division of Biosciences, Brunel University, London, UB8 3PH UK

**Keywords:** Kinetochores, Heterochromatin, Centromere, Condensin, Chromosome

## Abstract

In order to understand the three-dimensional structure of the functional kinetochore in vertebrates, we require a complete list and stoichiometry for the protein components of the kinetochore, which can be provided by genetic and proteomic experiments. We also need to know how the chromatin-containing CENP-A, which makes up the structural foundation for the kinetochore, is folded, and how much of that DNA is involved in assembling the kinetochore. In this MS, we demonstrate that functioning metaphase kinetochores in chicken DT40 cells contain roughly 50 kb of DNA, an amount that corresponds extremely closely to the length of chromosomal DNA associated with CENP-A in ChIP-seq experiments. Thus, during kinetochore assembly, CENP-A chromatin is compacted into the inner kinetochore plate without including significant amounts of flanking pericentromeric heterochromatin.

## Introduction

Centromeres were classically defined as the primary constriction of chromosomes—a region where paired sister chromatids are held tightly together until the onset of anaphase. Classic microscopic observations revealed that this was the region where mitotic chromosomes attached to the spindle microtubules. In the 1960s, electron microscopy first recognized that the surface of the centromere was the site of assembly of a button-like structure—the kinetochore—which formed the actual point of microtubule attachment (Luykx 1965; Brinkley and Stubblefield 1966; Jokelainen 1967). In subsequent years, studies by a great many laboratories have developed a still emergent picture of the kinetochore as one of the most wonderfully complex supramolecular structures found in cells (Maiato et al. 2004; Cheeseman and Desai 2008; Hori and Fukagawa 2012; Westhorpe and Straight 2013).

For years, there was an ongoing debate about the distinction (if any) between centromeres and kinetochores. Both terms were initially proposed to describe essentially the same aspect of chromosome structure and function, so this seemed liked a semantic discussion. However, in the post-genomic era of cloned and sequenced genomes, a meaningful distinction has emerged. It is now generally accepted that the underlying DNA sequence that defines the primary constriction of mitotic chromosomes is the centromere, whereas the proteinaceous structure that assembles on the surface of the centromeric chromatin is the kinetochore. The histone H3 variant CENP-A is a specific protein marker for centromeres (Earnshaw and Rothfield 1985; Earnshaw et al. 2013), whereas kinetochores are characterized by the presence of >100 different proteins, the earliest described being CENP-C (Earnshaw and Rothfield 1985; Saitoh et al. 1992).

At present, many groups are working to dissect the composition and structure of the kinetochore. Studies of the centromere have been more limited, in part due to its complex repetitive substructure. In budding yeast, the point centromere (Pluta et al. 1995) is defined by a 125 bp DNA sequence (Hegemann and Fleig 1993). In fission yeast and vertebrates, there is no single sequence that defines the centromere, with CENP-A being distributed across wide regions of DNA. We therefore coined the term “regional centromeres” to describe most centromeres outside budding yeast (Pluta et al. 1995).

With the advent of increasingly sophisticated molecular approaches, and particularly deep sequencing, it is now possible to begin to define the centromeric DNA in vertebrates by ChIP-seq in a few specialized instances. Detailed CENP-A mapping is not possible for most metazoan centromeres because they are comprised of hugely redundant families of repetitive DNA that have foiled efforts to create contiguous linear maps. The exceptions to this include human neocentromeres (du Sart et al. 1997; Barry et al. 1999; Burrack and Berman 2012), chromosome 11 of the horse (Wade et al. 2009), and chromosomes Z, 5, and 27 in chicken (Shang et al. 2010). All of those centromeres are assembled on unique sequence DNA, so that it is possible to exactly map the extent of the CENP-A domain.

Even though the CENP-A domain cannot be mapped precisely in humans, analysis of extended chromatin fibers has revealed that centromeres are comprised of intercalated domains of CENP-A chromatin and chromatin containing centromeric canonical histone H3 (Blower et al. 2002; Sullivan and Karpen 2004; Ribeiro et al. 2010; Sullivan et al. 2011). This observation lead to a working model in which the centromeric chromatin formed an amphipathic-like solenoid, with the CENP-A-containing surface at the chromosome exterior abutting the kinetochore and a H3-containing surface facing the interior of the chromosome (Sullivan and Karpen 2004). More recent super-resolution mapping of various kinetochore protein pairs (Joglekar et al. 2009; Wan et al. 2009; Varma et al. 2013) and observation of antibody-labeled chromatin fibers led to the proposal of a boustrophedon model of the kinetochore chromatin as multi-layered sinusoidally folded patch on the chromosome surface, rather than a helix embedded in it (Ribeiro et al. 2010).

One parameter that is essential in establishing detailed models for the folding of the centromeric chromatin fiber in the inner kinetochore is the amount of DNA in the assembled kinetochore. By this, we do not mean the amount of DNA occupied by CENP-A, as determined by ChIP-seq, but instead the amount of DNA that is folded into the three-dimensional functional mitotic kinetochore. This has not been measured, largely because the inner kinetochore is embedded in the centromeric chromatin, and cannot be distinguished from the surrounding DNA in compact mitotic chromosomes by conventional microscopy methods.

The aim of the present study was to take advantage of a chicken DT40 condensin mutant in which kinetochores undergo “excursions” during which they are occasionally stretched considerable distances from the surface of the centromeric chromatin (Ribeiro et al. 2009). Under those circumstances, the kinetochore is spatially isolated away from the rest of the underlying centromeric chromatin. We have used quantitative fluorescence to estimate the amount of DNA within the folded kinetochore domain as defined by CENP-H-GFP, expressed from its own promoter and exogenous CENP-A-GFP. This analysis revealed that DT40 kinetochores in their native conformation contain ~50–60 kbp of DNA, a number that is remarkably close to 50–150 kbp size of the CENP-A domain measured in human neocentromeres (Barry et al. 1999; Alonso et al. 2007) and the 41 ± 6 kbp size of the CENP-A domain measured by ChIP-seq for 12 chicken centromeres and neocentromeres (Shang et al. 2013). Thus, when kinetochores assemble on mitotic chromosomes, they do not incorporate significant amounts of pericentromeric DNA surrounding the core CENP-A domain.

## Results

### DNA content of a functional kinetochore

**Fig. 1:**
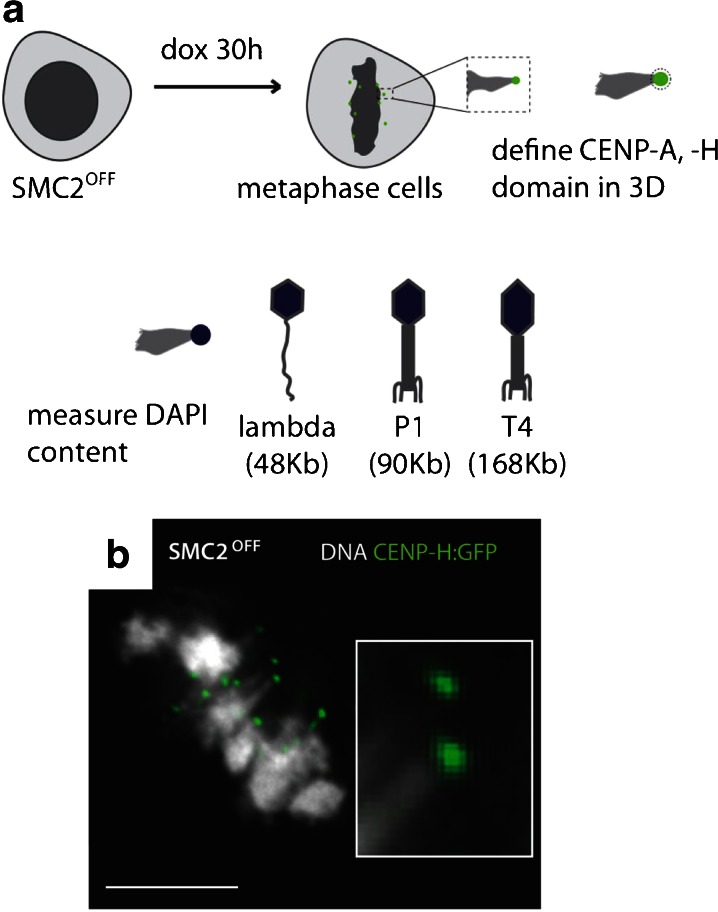
Quantification of DNA in a functional vertebrate kinetochore. **a** Experimental design used to measure kinetochore DNA content in SMC2^OFF^ cells and bacteriophages used as standards with known amounts of DNA. **b** Image of SMC2^OFF^ cell with several kinetochores undergoing “excursions”. Inset shows two kinetochores labeled with CENP-H-GFP with trailing chromatin (*scale bar* 5 μm)

The distribution of kinetochore proteins along the DNA has been mapped at vertebrate neocentromeres by a number of methods (Alonso et al. 2007; Shang et al. 2013; Saffery et al. 2003; Capozzi et al. 2008), but it is not known how much DNA is folded into a functional mitotic kinetochore. Our published studies have established that kinetochores of SMC2-depleted chicken DT40 cells are structurally and functionally normal (Ribeiro et al. 2009). Thus, condensin is not required to assemble a functional kinetochore in vertebrates. It is, however, required to regulate the compliance (stretchiness) of the pericentromeric heterochromatin, which acts as a spring linking the two sister kinetochores (Ribeiro et al. 2009; Gerlich et al. 2006; Jaqaman et al. 2010). Therefore, because condensin-depleted kinetochores are spatially well resolved from the surface of the primary constriction when undergoing “excursions” (Fig. 1a, b), they can be used to determine the DNA content of a functioning kinetochore in situ by quantifying the DNA within the compact CENP-H and CENP-A domains.

The strategy adopted here was to image metaphase kinetochores of SMC2-depleted cells that were expressing either CENP-H-GFP or CENP-A-GFP as three-dimensional image stacks, identify the volume occupied by CENP-H and CENP-A, and then ask how much DNA (stained with DAPI) was present within that volume (Fig. 1a). In each case, the DAPI staining was measured relative to a standard present in the same image. CENP-H was used in these experiments because the single copy CENP-H gene on the chicken Z chromosome had been labeled by knock-in of a GFP cDNA (Fukagawa et al. 2001). Thus, the fusion protein was expressed from its native promoter and was able to support life of the cells. In the case of GFP-CENP-A, the fusion protein was a transgene expressed in a stable cell line. Other work in human (Foltz et al. 2006) and DT40 (Shang et al. 2013) cells has shown that there appears to be a homeostatic regulation of CENP-A levels, such that when exogenous CENP-A is expressed, levels of the endogenous protein fall, so that overall CENP-A levels remain similar (Foltz et al. 2006). Furthermore, ChIP-seq analysis in chicken has shown that the size of CENP-A chromatin domains is not significantly increased following expression of exogenous CENP-A-GFP (Shang et al. 2013).

**Fig. 2:**
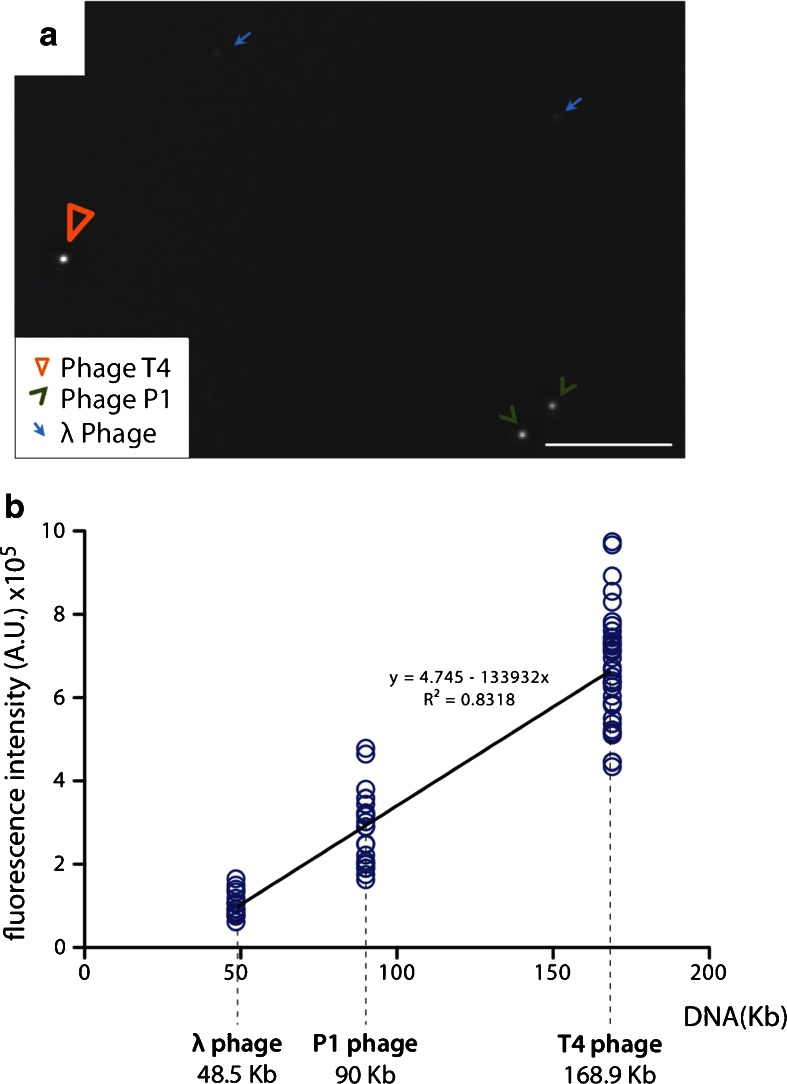
Three bacteriophages used as standards for the determination of DNA amount based on fluorescence intensity. **a** DAPI fluorescence image of a mixture of bacteriophages lambda, P1, and T4 in the same field of view (*scale bar* 5 μm). **b** Standard curve of fluorescence intensity (DAPI—*y* axis) and DNA amount in heads of bacteriophages lambda, P1, and T4 (*x* axis)

For use as a DNA standard, we initially used *S. cerevisiae* cells in G1 phase, but rapidly realized that the amounts of DNA in chicken mitotic kinetochores were far smaller than this. We therefore switched to the use of three bacteriophages, namely: T4 (genome size 168 kb), P1 (90 kb), and *λ* (48 kb) to calibrate the DNA quantification since their genome size is within the range of the DNA amounts in the kinetochore. When these bacteriophages were fixed, stained with DAPI, and mixed and imaged with the DeltaVision microscope (Fig. 2a), a linear correlation between DNA amount and intensity was obtained (Fig. 2b).

**Fig. 3:**
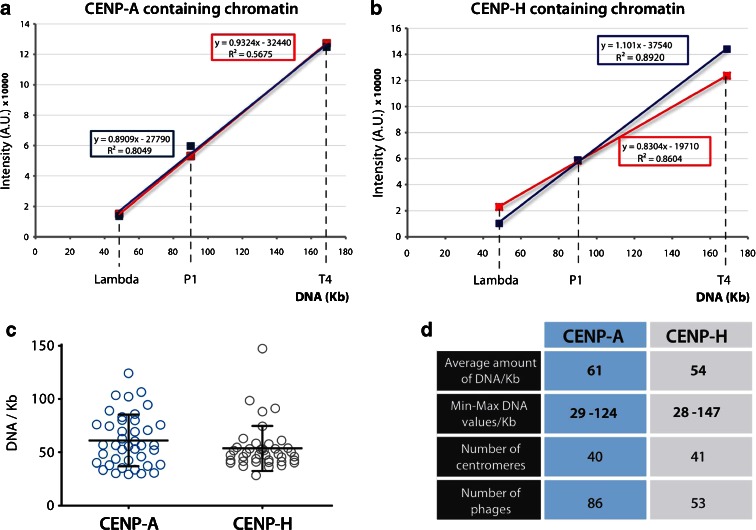
**a**, **b** Standard curves of the fluorescence intensity of DAPI-stained bacteriophages lambda, P1, and T4 mixed with SMC2^OFF^ cells expressing GFP-CENP-A (**a**) or CENP-H-GFP (**b**). *Blue* and *red standard lines* correspond to two independent experiments. These *standard curves* were used to plot the fluorescence intensity of the region of interest (ROI) corresponding to each of the GFP-tagged proteins and determine the amount of DNA. **c** Distribution of the amounts of DNA within CENP-A and CENP-H regions after plotting the values onto the standard curve obtained with the three internal bacteriophage references (graphs **a** and **b**). **d** Summary table showing values obtained from the quantification done in **c**

This calibration allowed us to quantify the amount of DNA in functional mitotic kinetochores. SMC2^ON/OFF^ cells expressing tagged endogenous (knock-in) CENP-H-GFP or exogenous GFP-CENP-A were mixed with bacteriophages and processed for image acquisition. We looked for cells in which one or more kinetochores had stretched polewards, away from the body of its chromosome and where we could also acquire the three different bacteriophages in the same field of view. In each case, the GFP-labeled protein was used to define a volume envelope for the kinetochore, and the amount of DAPI staining within that envelope was measured. Standard curves determined for the bacteriophages in each experiment were used to calibrate the DNA intensity values for the CENP-A or CENP-H-containing chromatin (Fig. 3a, b). It is worth emphasizing that these GFP-labeled kinetochores were functional at the time of fixation, as they were attached to microtubule bundles and exerting force towards the spindle poles. Furthermore, it is important to note that while the chromatin beneath the kinetochore was abnormally stretched, the kinetochores themselves appeared structurally normal at both the light and electron microscope levels (Ribeiro et al. 2009).

A measurement of ≥40 centromeres in each case revealed that compact CENP-A and CENP-H domains of functional SMC2-depleted kinetochores contained an average of 61 and 54 kb of DNA, respectively (range—28–147 kb, Fig. 3d). Because the kinetochores measured come from a number of different DT40 chromosomes, the observed range of DNA content could be in part due to size variation between kinetochores. However, a recent study in the Fukagawa lab has revealed that all of the kinetochores in DT40 cells are of similar size when measured with a variety of kinetochore markers (T. Hori and T. Fukagawa, personnal communication). Importantly, their measurements included kinetochores assembled on repetitive centromeres as well as those on nonrepetitive centromeres and on both micro and macro chromosomes. Thus, the most likely explanation for the variation is that because kinetochores are pulled away from the primary constriction to differing degrees, the amount of pericentromeric chromatin immediately adjacent to the kinetochore may vary.

## Discussion

In order to build a structural model of the vertebrate kinetochore, we need to know not only the length of chromosomal DNA occupied by CENP-A but also the way in which that DNA is folded in order to build a three-dimensional structure capable of withstanding the pulling forces generated by spindle microtubules. The earliest model proposed was for one or more stretches of chromatin superhelix with CENP-A facing the chromosome exterior and histone H3 facing the chromosome interior (Sullivan and Karpen 2004). We later proposed a multilayered patch-like model in which the chromatin fibers are folded in sinusoidal boustrophedons (Ribeiro et al. 2010). Importantly, neither of these models can exclude the possibility that stretches of chromatin not associated with CENP-A might be incorporated into the folded kinetochore-associated chromatin.

We have here combined gene targeting with deconvolution microscopy to estimate the amount of DNA in a fully functional vertebrate kinetochore. Our data indicate that functional chicken kinetochores, defined by the three-dimensional volumes occupied by CENP-A and CENP-H, contain 58 ± 23 kb of DNA. It is important to note that this amount is an estimate, as we have used a single DNA-binding dye (DAPI), which typically shows a preference for AT-rich DNA. Nonetheless, because the amount of DNA contained within each stretched centromere is influenced by the degree of stretching, which cannot be readily controlled, the values obtained can never be better than estimates.

The values measured in the present experiments show a remarkable similarity to the 41 ± 6 kb span of chromosomal DNA occupied by CENP-A on 12 newly generated chicken neocentromeres as measured by ChIP-seq (Shang et al. 2013). Interestingly, the amount of DNA in chicken kinetochores is close to values determined by chromatin immunoprecipitation (ChIP) for human neocentromeres, which range from 54 to 464 Kb [reviewed in (Marshall et al. 2008)].

Based on the present data as well as previous studies from our laboratory, we propose that the kinetochore is assembled from a single contiguous chromatin segment in which alternating CENP-A and H3 domains are folded into a planar sinusoidal patch, or boustrophedon. We have previously argued that the boustrophedon is composed of several layers held together by structural crosslinks that depend upon CENP-C for their formation and stability (Ribeiro et al. 2010). Present studies from our group aim to determine whether other kinetochore proteins are also required to stabilize the multilayered kinetochore structure and determine the number of nucleosomes per layer.

In the future, other super-resolution microscopy approaches will hopefully combine with genome three-dimensional mapping approaches (e.g., 3C, 4C, etc. (Dekker et al. 2013; Naumova et al. 2013)) to permit the development of more accurate models for the folding of the chromatin fiber in centromeres.

## Material and methods

### Cell culture

The SMC2 conditional knockout cell line, SMC2:CENP-H:GFP and SMC2:GFP:CENP-A were cultured as previously described (Ribeiro et al. 2009; Vagnarelli et al. 2006).

### Cell and phage fixation and staining

Condensin was depleted from SMC2 ^ON/OFF^ conditional knockout cells expressing CENP-H-GFP or GFP-CENP-A by 30-h exposure to doxycycline, following which, cells were fixed with paraformaldehyde at 4 %. T4, P1, and *λ* bacteriophages were kindly provided by Dr. Noreen Murray. Fifty microliter of each phage were washed in SM buffer (5 M NaCl, 1 M MgSO_4_, 1 M Tris–HCl pH 7.5), fixed in 4 % PFA-SM for 5 min, and resuspended in 30 μl of Vectashield with 1.5 μg/ml DAPI for 20 min. For the quantification analysis, 2 μl of the stained phages in Vectashield were used to mount the slides with SMC2^OFF^ cells. This concentration was determined empirically to give a sufficient number of bacteriophages so that each field of view containing a kinetochore undergoing an excursion would have a few bacteriophages in the background.

### Quantification of DNA using Image-Pro Plus

SMC2^ON/OFF^ immunostaining image stacks were acquired using a microscope (IX-70; Olympus) with a charge-coupled device camera (CH350 or HQ; Photometrics) controlled by DeltaVision SoftWorx (Applied Precision, LLC) and a 100× S Plan Apochromat NA 1.4 objective using a Sedat filter set (Chroma Technology Corp.) and running at RT. We selected for imaging metaphase aligned cells where kinetochores were clearly resolved from the rest of the chromatin (images of 1,024 × 1,024 pixels in z-stacks with a 200 nm step size). In the same field of view, phages were also acquired and used to define a standard curve between florescence intensity and DNA amount. Image stacks were deconvolved, and maximum projections were generated using SoftWorx. All files were saved as TIFF files and exported to Photoshop (Adobe) for final presentation. Levels were adjusted similarly for each experimental dataset to lower nonspecific background haze using the standard Photoshop adjust levels tool.

After deconvolution, we measured the DAPI content in the volume occupied by CENP-A or CENP-H. Image planes where kinetochore “excursions” were observed were sum-quick projected. Single planes where phages were observed were selected for each image acquired. For the phage, the area was defined in the DAPI channel. All the images were saved as TIFF files.

For the phage images, the single TIFF file was opened in Image-Pro Plus. Regions of interest (ROI) covering the phage area were selected by hand, and sum intensity value was annotated. The same ROI was moved between 5 and 10 pixels, and the intensity was annotated as background. The intensity value was determined by subtracting the background value from the sum value. The intensity values for all phages in each experiment were entered in GraphPad Prism (GraphPad Software, Inc. CA, USA) where a XY graph was plotted and the best-fit linear regression calculated representing the linear standard of amount of DNA versus intensity. For the SMC2^OFF^ cells, separated TIFF files of the 488 and 528 channels were opened in Image-Pro Plus (Media Cybernetics, Inc., Bethesda, USA). Using the 528-nm image, a ROI covering the area covered by the pulled centromere was selected. This ROI was copied and loaded into the 488-nm file where the sum value of intensity was determined. Again, the background value was determined by moving the ROI 5–10 pixels away from the metaphase plate. The intensity of the DAPI channel was determined by subtracting the background. The absolute amount of DNA was calculated according to the equation of the previously determined best-fit linear regression.

The line profile was drawn in ImageJ (Research National Institute of Mental Health, Bethesda, USA) values exported to Excel (Microsoft, Redmond, WA) and graphs of each channel superimposed.

## Abbreviations

CENP: Centromere protein
ChIP: Chromatin immunoprecipitation
GFP: Green fluorescent protein
H3: Histone H3
SMC2: Structural maintenance of chromosomes protein 2
